# Geospatial analysis of tuberculosis incidence in relation to socio-economic and environmental indicators in Romania

**DOI:** 10.3389/fpubh.2025.1711489

**Published:** 2025-12-09

**Authors:** Jenna Zabroski, Daniel Peptenatu, Beatrice Mahler, Amr Soliman, Andreea Karina Gruia, Alexandra Grecu, Ioana Munteanu, Aurel Bǎloi

**Affiliations:** 1 Cancer Epidemiology Education in Special Populations (CEESP), New York, NY, United States; 2 Faculty of Interdisciplinary Studies, University of Bucharest, Bucharest, Romania; 3 Interdisciplinary Center for Advanced Studies (CISA-ICUB), University of Bucharest, Bucharest, Romania; 4 Faculty of Geography, University of Bucharest, Bucharest, Romania; 5 Carol Davila University of Medicine and Pharmacy, Bucharest, Romania; 6 Marius Nasta Institute of Pneumology, Bucharest, Romania; 7 Department of Community Health and Social Medicine, School of Medicine, City University of New York, New York, NY, United States; 8 Faculty of Administration and Business, University of Bucharest, Bucharest, Romania; 9 Faculty of Medicine, “Titu Maiorescu” University of Bucharest, Bucharest, Romania; 10 Graphit Innovation Factory, Drobeta-Turnu Severin, Romania

**Keywords:** tuberculosis, air pollution, living conditions, education levels, exploratory analysis, geospatial analysis, spatial epidemiology

## Abstract

**Introduction:**

Tuberculosis (TB) is a global public health burden. Romania experiences some of the highest rates of TB compared to other European countries. TB incidence is spatially clustered throughout Romania, and the reasoning behind this is likely linked to various environmental and socio-economic factors. This study aimed to investigate the spatial distribution of TB incidence per 1,000 population between 2015 and 2021 and examined its correlation with particulate matter (PM2.5) levels, living conditions, and level of educational indicators.

**Methods:**

TB incidence data from 2015 to 2021 aggregated at the level of 3,181 administrative territorial units (UATs) in Romania were analyzed. Pearson correlation coefficients examined linear associations among total TB incidence and exploratory indicators. A Principal Component Analysis (PCA) was conducted due to the presence of multicollinearity in the Pearson correlation matrix, which grouped key housing indicators into one hybrid living condition index (PCA1). Bivariate Moran's I analyses assessed localized spatial clustering between TB incidence and two exploratory indicators: PM2.5 levels and PCA1. A spatial lag regression model accounted for spatial dependence between TB incidence and three explanatory predictors: PM2.5 levels, the percentage of the population that is illiterate, and PCA1. All significance tests were conducted at a threshold of *p* < 0.05.

**Results:**

TB incidence is spatially clustered in Romania. PM2.5 levels (*r* = 0.261, *p* < 0.0001) and PCA1 (*r* = 0.338, *p* < 0.0001) are positively and significantly associated with TB incidence. Illiteracy showed no significant association with TB incidence. The spatial lag model confirmed spatial autocorrelation (Rho = 0.436, *p* < 0.0001) and explains 28.4% of the variation in TB incidence across Romania.

**Discussion/Conclusion:**

This is the first geospatial study in Romania that explores the link between TB incidence and exploratory indicators of air pollution, living conditions, and education level using epidemiological data obtained in the national tuberculosis surveillance and control program. Greater exposure to air pollution and worsened living conditions are correlated with higher TB incidence rates. Policymakers should highlight the need for geographically targeted interventions to improve TB control and screening in Romania.

## 1 Introduction

According to the World Health Organization (WHO), tuberculosis (TB) is a globally widespread infection and is one of the world's leading infectious diseases. It is an infectious disease caused by the bacterium *Mycobacterium tuberculosis*. It mainly affects the lungs but can also impact other areas of the body. There are millions of new cases and deaths caused by TB each year. An estimated 10.8 million people globally fell ill from TB in 2023, equivalent to a global incidence of 134 out of 100,000 (1, 2). Analysis of data from the European Center for Disease Prevention and Control (ECDC) shows that in Eastern Europe, tuberculosis remains a significant public health problem, with Romania reporting some of the highest incidence rates of tuberculosis. Romania accounted for almost a quarter of all TB cases reported in 2023 among the 29 EU/EEA countries. In 2023, Romania also reported the highest age-specific rates of tuberculosis among children, with 19.7 cases per 100,000 inhabitants in those aged 0–4 years. The frequency of tuberculosis in Romania differs from one region to another, which makes geospatial research important for clarifying the complex relationships between the determinants of pathology (3–5).

Geospatial studies on the spatial distribution of tuberculosis in Romania have demonstrated a marked spatial heterogeneity in the distribution of pathology. Thus, the areas of concentration of the pathology are identified, the clusters being present in Oltenia and the south of the Muntenia regions, sporadically in Moldova and Dobrogea and some areas in the Banat region. This spatial heterogeneity suggests that spatial clustering and geospatial analysis are important factors in understanding the epidemiology of tuberculosis in Romania. Problematic foci of tuberculosis incidence have been identified; However, it is not clear why this space grouping exists. This analysis suggests that heterogeneity is due to individual factors associated with disease risk, environmental conditions, social marginalization, and limited access to health services. The spatial grouping reflects epidemiological patterns in disease incidence, but also suggests that other social and environmental determinants may also affect TB rates (5).

The literature highlights numerous factors that have been linked to the increase in tuberculosis rates, among the most important being population density, lack of running water, housing quality, geographical isolation, poverty and unemployment (6–15). Added to this are air pollution, access to healthcare, overcrowding, poor ventilation, education levels, and lack of medical infrastructure (16–20). The spatial distribution of tuberculosis highlighted the concentrations of high tuberculosis values, the present research being oriented toward the correlation between the incidence of tuberculosis in Romania, between 2015 and 2021, and three exploratory areas of interest: air pollution, living conditions and level of education.

Socio-economic status plays a key role in shaping health outcomes and is a determinant of TB incidence. The literature indicates that poverty and low income are some of the most important factors in the incidence of tuberculosis (21–28). Poor housing quality, characterized by overcrowding, inadequate sanitation, lack of running water, toilets, and heating, and limited access to health infrastructure, is also correlated with a higher incidence of tuberculosis (29–31). In Romania, the increased incidence of tuberculosis has been linked to socio-economic factors such as standard of living, quality of life, psychological suffering, social isolation, so TB affects communities where these factors have cumulative effects (16, 28, 32–34).

Lower levels of education and literacy are associated with delays in the diagnosis and treatment of TB (35–37). The literature has conflicting findings about the association between the level of education and TB incidence. Some studies suggest that lower levels of education are correlated with an increased incidence of TB, while some studies find the opposite (25, 37–40). This is not to say that education influences other behaviors, including awareness and engaging in healthy behaviors. Rather, studies suggest that education and literacy are tied to other factors like income and access to utilities (37, 41–43).

Air pollution is becoming increasingly recognized in the literature as a risk factor for tuberculosis. Long-term exposure to PM2.5 and PM10 is consistently associated with a higher risk of active tuberculosis (17, 18, 20, 44–48). Fine particles can penetrate deep into the lungs, which causes inflammation and can affect the pulmonary immune defenses (49, 50). In addition, exposure to both nitrogen dioxide and sulfur dioxide has been linked to increased incidence of tuberculosis (20, 44–47). In terms of PM2.5 exposure, the risk of TB increased by 6%−12% for every 10 μg/m3 of exposure (20, 44–47). Urban areas and locations closer to industrialized areas tend to have higher levels of PM2.5, which could contribute to the local incidence of tuberculosis (51).

According to our research strategy and design, this study is the second of a planned trilogy: first identifying TB high-risk areas, exploring underlying factors, and a third paper forthcoming to assess the impact and action of interventions. In the previous exploratory spatial analysis study, the main TB hotspots were identified along with outlier regions in Romania (5). This work mapped the foundation for the geographical distribution of TB cases, but did not examine any underlying factors that drove spatial patterns in TB incidence in Romania.

Building on this, the current study aimed to determine spatial distribution of TB incidence in Romania between 2015 and 2021 through three exploratory areas: living conditions, education level and air pollution. The study examined whether TB geospatial clusters in Romania are sensitive to different degrees to different living conditions (e.g., dwellings with bathroom, toilet, sewerage, heating, etc.), education level (percentage of illiterate population) and air pollution (PM2.5 levels). There is little research that has been done on the link between tuberculosis incidence and these geospatially exploratory variables in Romania. These specific variables are of interest because they are the indicators where data are publicly available in Romania.

The analysis of the incidence of tuberculosis in Romania requires an integrated approach, capable of capturing the complex interaction between the social determinants of health and environmental factors. The present study responds to the requirements by bringing together datasets from official sources, in order to highlight how socio-economic conditions and air quality influence the geographical distribution of the disease. The epidemiological data on tuberculosis were taken from the National Program for Tuberculosis Prevention, a reference source that ensures continuous and standardized monitoring of the situation at national level. This information was correlated with socio-economic indicators collected by the Territorial Observatory of Romania, an online public platform managed by the Ministry of Regional Development and Public Administration, which centralizes essential data for assessing territorial development and interregional disparities (52). In addition to these data, the analysis also includes the concentrations of fine particulate matter PM2.5, obtained through the Air Quality Life Index (AQLI), a tool developed by the Energy Policy Institute of the University of Chicago, aimed at quantifying the impact of air pollution on health and life expectancy (53). The integration of these sources at the level of administrative-territorial units allows the application of exploratory and statistical geospatial methods to identify correlations and spatial models describing the relationship between tuberculosis incidence, living conditions, education level and air pollution. This approach is justified by the fact that tuberculosis, although it has a well-defined infectious etiology, is deeply influenced by structural factors such as poverty, limited access to health services, low level of education and chronic exposure to air pollutants. Therefore, an analysis that integrates these parameters not only describes the epidemiological reality, but also contributes to the fundamentals of evidence-based public policies, oriented toward reducing health inequalities and improving the condition.

This research aimed to fill the current knowledge gaps in Romania regarding the impact of living conditions, education level and environmental pollution on TB incidence, as well as their geospatial distribution. By identifying spatial hotspots for the spread of the disease in Romania and analyzing it at the level of all administrative-territorial units, this allows a deeper understanding of the link between tuberculosis incidence and living conditions, education level and air pollution. Geospatial analysis is essential in understanding the incidence of tuberculosis, so the spatial model of TB can assist decisions within public policy on the management of the disease.

## 2 Materials and methods

### 2.1 Unit of analysis and observation

This research was conducted at the level of all 3,181 administrative territorial units (UAT) in Romania. Data interpretations are made at the UAT, county, and development region levels. In Romania, UATs are the smallest form of political-administrative organization of communities, operating within a territorial system with specialized decision-making functions, regulated by a mayor and a local council. By using the UAT level of analysis in this research, it allows for a granular approach to geospatially understand how TB incidence potentially correlates with environmental and socio-economic indicators in Romania. The National Agency provided the shapefile of Romania at the UAT level of analysis for Cadastre and Real Estate Publicity on the open data website (https://data.gov.ro/) (54). This is coordinated by the General Secretariat of the Romanian Government, and the official nomenclature of administrative territorial units in Romania is called SIRUTA.

### 2.2 Data sources (data collection)

This study uses data from three primary data sources, aggregated at the level of UATs in Romania.

Incidence of tuberculosis data was obtained from the Marius Nasta Institute of Pneumophthisiology, the unit that coordinates the National Tuberculosis Prevention, Surveillance and Control Program in Romania. This represents the consolidated national database of all tuberculosis cases registered annually in Romania. The database contained information annually from 2012 to 2021, at the individual level, with no access to personal identifiers, and has been used in compliance with GDPR policies. All new cases and relapses were recorded annually to determine the variable number of registered cases. The data were then aggregated at the UAT level of analysis, based on the analysis dimensions of sex and the affected organ (pulmonary vs. non-pulmonary). For geospatial analyses, data aggregated by UAT from 2015 to 2021 were used in the analyses. The annual number of cases, disaggregated by sex and the affected organ, is provided in the dataset. The dataset also includes yearly and total incidence rates of TB per 1,000 population. Total incidence rates per 1,000 population are stratified by sex and affected organ. In order to report the number of new cases and relapses to the population, annual data at the UAT level, national demographic statistics published on the official website of the National Institute of Statistics (Tempo online) was obtained. Population data to calculate incidence is based on data published in 2015 and will be used as the reference year. The total incidence of TB between 2015 and 2021 is the dependent variable in the spatial lag regression model.

While initial data records for TB incidence exist from 2012, consistent data at the administrative-territorial unit (UAT) level necessary for spatial analyses in this analysis were only available from 2015–2021. This informed our decision to focus quantitative spatial and regression analyses during the time period of 2015–2021. Trend visualizations include data since 2012.

In the analysis, the annual values of TB new cases and relapses were used to calculate the total incidence from 2015 to 2021. Incidence was calculated per 1,000 population in Romania and was calculated using the following formula (1):


$$\begin{array}{l} \text{Incidence }=\frac{\text{Number of New Cases and Relapses x }1,000}{\text{Total Population in }2015} \end{array}$$

The Air Quality Life Index (AQLI), developed by the Energy Policy Institute at the University of Chicago, is sourced from yearly data on particulate matter (PM2.5) concentrations in Romania, aggregated by UAT. PM2.5 levels were collected between 2015 and 2021 to match the TB incidence data period. Average levels of PM2.5 were calculated between 2015 and 2021 and used in the analysis. The AQLI collects information on PM2.5 concentrations according to van Donkelaar by using satellite-derived annual ambient PM2.5 concertation estimates. For more specific information, refer to the methodology described in van (55). Average PM2.5 levels at the UAT level spatially align with the TB incidence data.

Data on six different socio-economic indicators were retrieved from the Territorial Observatory platform. This is an online portal of data that is operated by the Ministry of Regional Development and Public Administration in Romania. This interactive platform provided publicly available data that characterizes socio-economic dynamics and disparities across Romania. Data on socio-economic indicators were provided at the UAT level, allowing for the assessment of the potential association with TB between 2015 and 2021. All data were integrated and processed in the Q-GIS platform into a single GeoPackage. Microsoft Office Excel 2019 software was used to apply descriptive analysis techniques. Q-GIS was used to capture the geospatial descriptive statistics of TB in Romania. GeoDa was used for all geospatial statistical analysis techniques and regression models. SAS Studio was used to generate Pearson correlation matrices.

### 2.3 Indicators/variables

This analysis relied on several indicators, all of which were aggregated at the UAT level. Data on tuberculosis outcomes, environmental exposure measures, and socio-economic determinants were all assessed.

The primary outcome was the total incidence of tuberculosis (TB) per 1,000 population between 2015 and 2021. The total incidence values were calculated using data aggregated at the UAT level, provided by the National Institute of Statistics. The dataset was further disaggregated by sex and the affected organ, although only geospatial descriptive statistics were provided using the Q-GIS platform. Geospatial statistical analyses focused on the total incidence of TB.

This analysis is the second of a paper previously published in *GeoHealth, titled Spatio-Temporal Pattern of Tuberculosis Distribution in Romania and Particulate Matter Pollution Associated with Risk of Infection [will be referenced as the GeoHealth publication for the rest of the analysis]*. This paper assesses the spatiotemporal pattern of TB distribution in Romania and particulate matter pollution associated with the risk of infection (5). The previous study identified the main geographical areas of tuberculosis concentration in Romania, which has among the highest TB rates in Europe (5). In the local spatial autocorrelation of the incidence of TB per 1,000 population cumulated between 2015 and 2021, ~30% of the UATs in Romania fall into spatial clusters (5). 952 UATs are considered significant at a *p* = 0.05 threshold. These outcomes are taken into consideration for further analyses (5).

PM2.5 levels were measured at each UAT. In the previous *GeoHealth* publication, local spatial autocorrelation analyses were also applied to PM2.5 levels. The average PM2.5 levels were determined at the territorial administrative level for the 2015–2021 interval, and local spatial autocorrelation was applied. This analysis with PM2.5 levels is a continuation of the findings from the paper published in *GeoHealth* (5).

Data on six socio-economic indicators were collected from the Territorial Observatory Platform. All data was aggregated at the UAT level. These indicators include the percentage of the population that is illiterate, the percentage of houses without heating, the percentage of dwellings connected to the sewer, the percentage of dwellings with a bathroom, the percentage of dwellings with a toilet, and the percentage of dwellings with running water. All of this data is publicly available and used in further geospatial statistical analysis.

This analysis focused specifically on living conditions, education level, and air pollution as key indicators as there was consistent, publicly available data at the UATlevel in Romania. Although other predictors, such as HIV infection and drug use are important risk factors for TB, relevant data is not available for inclusion in this analysis.

### 2.4 Research question

(1) Why does the total incidence of tuberculosis between 2015 and 2021 display spatial clustering in Romania?
(2) How do living conditions, level of education, and environmental exposure, spatially correlate with tuberculosis incidence in Romania between 2015 and 2021?
(3) To what extent does spatial dependency affect the total incidence of TB in Romania between 2015 and 2021?

### 2.5 Statistical analysis techniques

To statistically explore the spatial relationship between tuberculosis incidence from 2015 to 2021 and environmental/socio-economic indicators, various statistical analysis techniques were employed. All statistical significance tests are performed at a significance level of 0.05.

The initial assessment of the data involved comparative choropleth mapping with the same legend to geographically visualize patterns in total TB incidence and compare TB incidence disaggregated by sex and the affected organ. All choropleth maps were formulated in the Q-GIS platform at the UAT level of analysis.

A Pearson correlation matrix was constructed using SAS Studio to evaluate the linear association between the total incidence of TB between 2015 and 2021 (i_20152021) and potential explanatory variables. These variables include average PM2.5 levels between 2015–2021 (aqli_pm25), the percentage of illiterate population (iillit_pop), the percentage of the population with no heating (noheating), the percentage of dwellings with a toilet (d_toilet), the percentage of dwellings with running water (d_runwater), the percentage of dwellings with a bathroom (d_bathroom), and the percentage of dwellings to a sewer (d_sewer). Pearson correlation coefficients (*r*) and significance levels (*p*-values) were assessed to determine the potential associations that could be used in further geospatial analyses.

The incidence of TB was positively correlated with PM2.5 levels (*r* = 0.261, *p* < 0.0001) and the percentage of the population that is illiterate (*r* = 0.102, *p* < 0.0001; Table 1). There is a strong perception in the community of specialists that TB incidence is correlated with lack of literacy which is why this indicator will be used in further analyses. TB incidence showed no significant correlation with the percentage of houses with no heating (*r* = 0.014, *p* = 0.44), and therefore this indicator was not included in further geospatial analyses. TB incidence showed negative correlation with other socio-economic indicators, particularly percentage of dwellings with a toilet (*r* = −0.313, *p* < 0.0001), percentage of dwellings with running water (*r* = −0.244, *p* < 0.0001), percentage of dwellings with a bathroom (*r* = −0.350, *p* < 0.0001), and percentage of dwellings with a sewer (*r* = −0.363, *p* < 0.0001). Strong multicollinearity was observed among the socio-economic indicators themselves, which showed a negative correlation with TB incidence. This suggests that these indicators overlap in their spatial correlation with TB incidence in Romania.

**Table 1:** Pearson correlation matrix of total incidence of TB between 2015-2021 (i_20152021) and potential explanatory variables (aqli_pm25, illit_pop, noheating, d_toilet, d_runwater, d_bathroom, d_sewer).

**Matrix**	**i_20152021 total incidence of TB between 2015–2021**	**aqli_pm25 average PM2.5 levels between 2015–2021**	**illit_pop the percentage of illiterate population**	**Noheating the percentage of the population with no heating**	**d_toilet the percentage of dwellings with a toilet**	**d_runwater the percentage of dwellings with running water**	**d_bathroom the percentage of dwellings with a bathroom**	**d_sewer the percentage of dwellings to a sewer**
i_20152021 Total incidence of TB between 2015–2021	1.00000	0.26084	0.10207	0.01364	−0.31268	−0.24381	−0.35028	−0.36283
		<0.0001	<0.0001	0.4420	<0.0001	<0.0001	<0.0001	<0.0001
aqli_pm25 average PM2.5 levels between 2015–2021	0.26084	1.00000	0.04364	0.00927	−0.06324	−0.12240	−0.09886	−0.15051
	<0.0001		0.0138	0.6013	0.0004	<0.0001	<0.0001	<0.0001
illit_pop the percentage of illiterate population	0.10207	0.04364	1.000	−0.04934	−0.26587	−0.16649	−0.28191	−0.28930
	<0.0001	0.0138		0.0054	<0.0001	<0.0001	<0.0001	<0.0001
Noheating the percentage of the population with no heating	0.01364	0.00927	−0.04934	1.00000	0.14519	0.11088	0.13855	0.12475
	0.4420	0.6013	0.0054		<0.0001	<0.0001	<0.0001	<0.0001
d_toilet the percentage of dwellings with a toilet	−0.31268	−0.06324	−0.26587	0.14519	1.00000	0.72505	0.97170	0.93060
	<0.0001	0.0004	<0.0001	<0.0001		<0.0001	<0.0001	<0.0001
d_runwater the percentage of dwellings with running water	−0.24381	−0.12240	−0.16649	0.11088	0.72505	1.00000	0.75393	0.79932
	<0.0001	<0.0001	<0.0001	<0.0001	<0.0001		<0.0001	<0.0001
d_bathroom the percentage of dwellings with a bathroom	−0.35028	−0.09886	−0.28191	0.13855	0.97170	0.75393	1.00000	0.96870
	<0.0001	<0.0001	<0.0001	<0.0001	<0.0001	<0.0001		<0.0001
d_sewer the percentage of dwellings to a sewer	−0.36283	−0.15051	−0.28930	0.12475	0.93060	0.79932	0.96870	1.00000
	<0.0001	<0.0001	<0.0001	<0.0001	<0.0001	<0.0001	<0.0001	

Pearson correlation coefficients, N = 3,181; Prob > |r| under H0 Rho = 0.

To ensure the multicollinearity between the percentage of dwellings with a toilet, the percentage of dwellings with running water, the percentage of dwellings with a bathroom, and the percentage of dwellings with a sewer is taken into consideration, a Principal Component Analysis (PCA) was performed between these variables in the GeoDa platform (Table 2). PCA was performed using the eigen method. PCA techniques reduce the dimensionality of multiple correlated housing living condition indicators, addressing the issue of multicollinearity through Pearson correlation analyses. Initial correlation matrices indicated strong intercorrelations among these variables, so adding a PCA analysis increases the reliability of the regression model.

**Table 2:** Principal component analysis of living condition indicators.

**Statistic**	**PC1**	**PC2**	**PC3**	**PC4**
Eigenvalue	3.58	0.34	0.06	0.02
Proportion of variance explained	0.90	0.08	0.02	0.01
Cumulative proportion	0.90	0.98	1.00	1.00
**Variable**				
d_sewer (loading)	−0.52	0.12	0.73	0.43
d_bathroom (loading)	−0.52	0.30	0.05	−0.80
d_toilet (loading)	−0.51	−0.36	−0.66	0.42
d_runwater (loading)	−0.45	−0.88	−0.16	−0.04

PCA allows for the summary of these socio-economic indicators into one living condition hybrid indicator (PCA1), which will then be used in all further spatial and regression analyses. PCA1 had an eigenvalue of 3.58 and accounted for nearly 90% of the total variance among the four indicators. All four variables loaded negatively onto PCA1, suggesting that these indicators, combined, capture socio-economic housing and infrastructure together, rather than as individual indicators. According to the Kaiser criterion (eigenvalue > 1) and the 95% explained variance threshold, the first component will be used in further analyses. PCA1 will be used in further spatial and regression analyses as one composite indicator. PCA2 and other components were not included in further analyses as these did not contribute substantial variance and lacked explanatory power to be included in the spatial lag model. PCA1 alone provides sufficient information to be used as a hybrid indicator and accurately capture living conditions in future analyses.

Bivariate Moran's I analyse were conducted to explore the spatial autocorrelation between tuberculosis incidence (2015–2021) and two explanatory variables: PM2.5 concentrations and PCA1. All bivariate analyses were conducted at the UAT level of analysis in GeoDa. These analyses identify spatial clusters where high or low levels of TB incidence are statistically associated with high or low values of the explanatory variable in neighboring areas.

**Figure 1:**
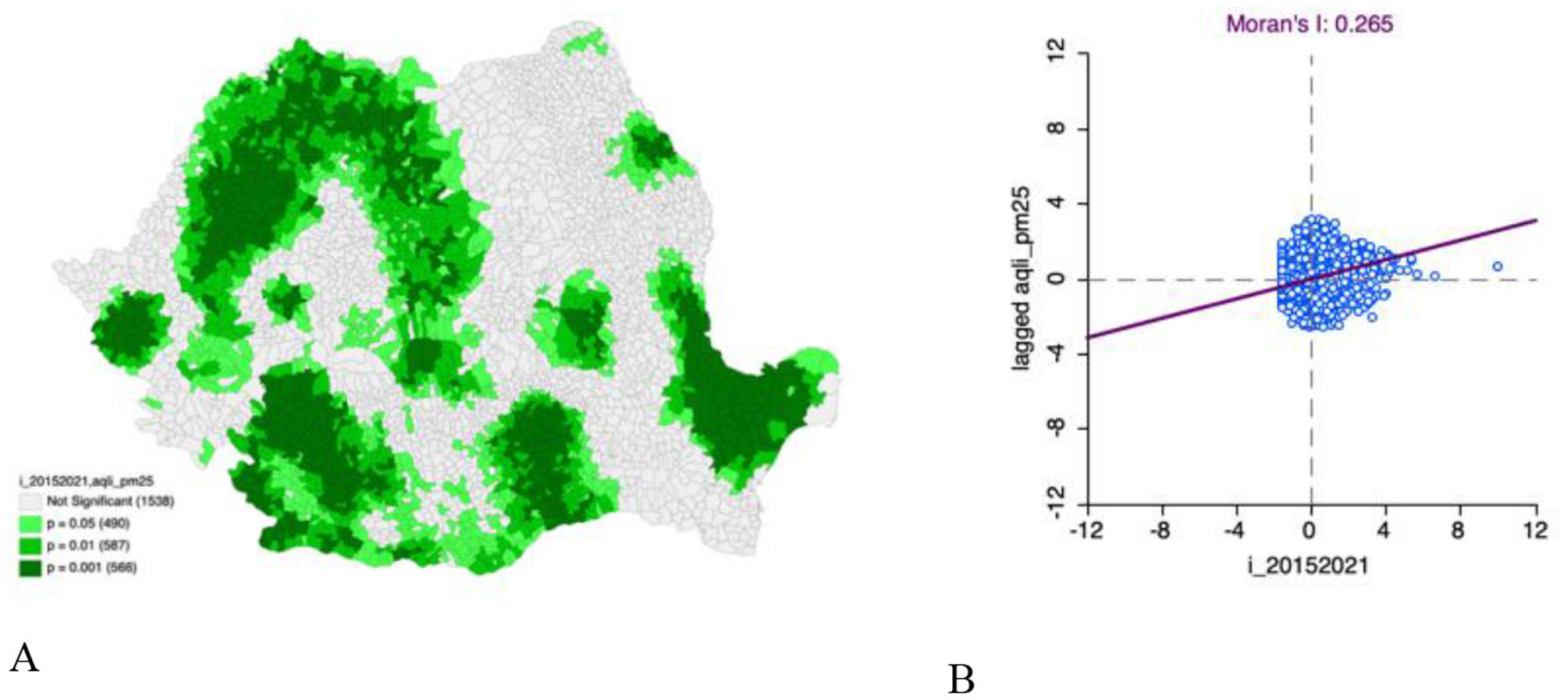
**(A)** Bivariate Moran's I: total incidence of TB (2015–2021) and PM2.5 levels significance map; **(B)** Bivariate Moran's I: total incidence of TB (2015–2021) and PM2.5 levels scatter plot.

Previous research has highlighted strong links between high TB incidence values and PM2.5 pollution (Moran I = 0.96) (5). Figure 1A shows the significance map of the bivariate Moran analysis between total TB incidence (2015 to 2021) and PM2.5 levels. It highlights which UATs show statistically significant local spatial autocorrelations between tuberculosis incidence and PM2.5 levels. A significant number of UATs in Romania, 1,643, have statistical significance in the bivariate analysis between the total incidence of TB in the period 2015–2021 and the level of PM2.5. The main representative clusters are in the South-West Development Region, Oltenia, Eastern Muntenia and Maramure? regions. Some clusters are also being revalidated in parts of the regions of Moldova and Transylvania. In the regions of Timisoara and Iasi there are two new clusters that are sensitive to the bivariate relationship between TB incidence and PM2.5 levels. Figure 1 shows the scatter plot between TB incidence and PM2.5 levels. Moran's global statistic I is 0.265, suggesting a moderate positive spatial autocorrelation between these variables. Areas with a high incidence of tuberculosis tend to be located near areas with high levels of PM2.5, and areas with a low incidence of tuberculosis tend to be located near areas with low levels of PM2.5. Spatial grouping is evident between TB incidence and PM2.5 levels.

**Figure 2:**
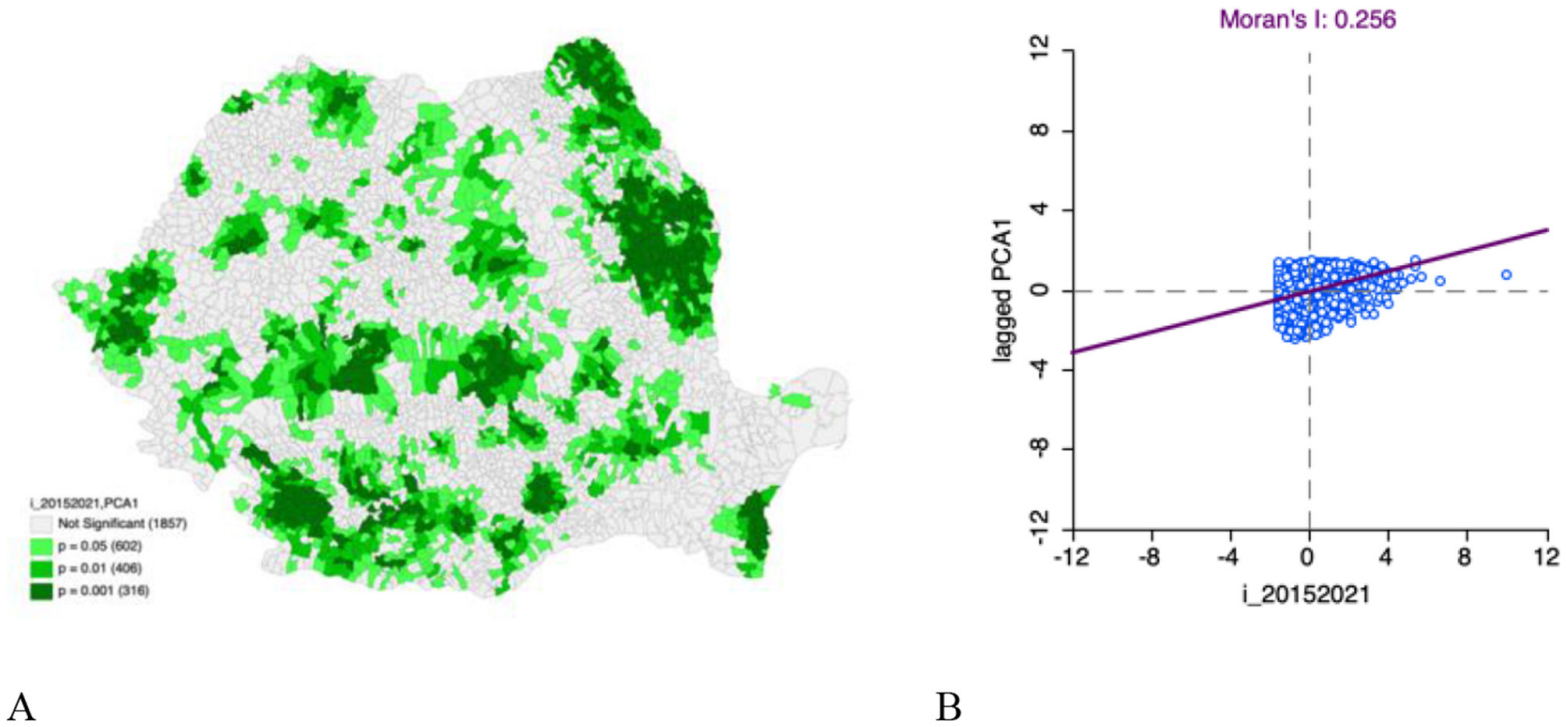
**(A)** Bivariate Moran's I: total incidence of TB (2015–2021) and PCA1 significance map; **(B)** Bivariate Moran's I: total incidence of TB (2015–2021) and PCA1 scatter plot.

The significance map of the bivariate Moran analysis between the total incidence of TB from 2015 to 2021 and PCA1, a composite living condition indicator derived from PCA, is shown (Figure 2A). It highlights which geographic UATs show statistically significant local spatial correlations between TB incidence and PCA1. Statistically significant areas suggest a spatial relationship between TB incidence and PCA1. There are 1,324 statistically significant UATs in Romania in the bivariate analysis between total incidence of TB between 2015–2021 and PCA1. Clusters are significantly revalidated in the central, southern, northeast, and slightly in the western part of Romania. These regions of Romania have significant positive spatial autocorrelation of PCA1.

The scatter plot depicting the relationship between TB incidence and PCA1 is provided (Figure 2B). The Bivariate Moran's I statistic is 0.256, suggesting a moderate positive spatial autocorrelation between these variables. Areas with high TB incidence tend to be geographically clustered near areas with high percentages of the PCA1 hybrid living conditions indicators, and areas with low TB incidence tend to be located near areas with low percentages of the PCA1 hybrid living condition indicators. Spatial clustering is apparent between TB incidence and PCA1 and reinforces the relationship between TB incidence and living conditions in Romania.

To model spatial dependence and further assess the relationship between TB incidence and predictor variables, specifically PM2.5 levels, PCA1, and the percentage of the population that is illiterate, a spatial lag regression analysis was performed. A spatial lag regression model accounts for the influence that neighboring UATs have on each observation. This addresses spatial autocorrelation in the analysis of TB incidence. There is a potential for heteroskedasticity to be present in this model; therefore, the residuals were further investigated using a residual choropleth map and a Global Moran's I scatter plot.

**Figure 3:**
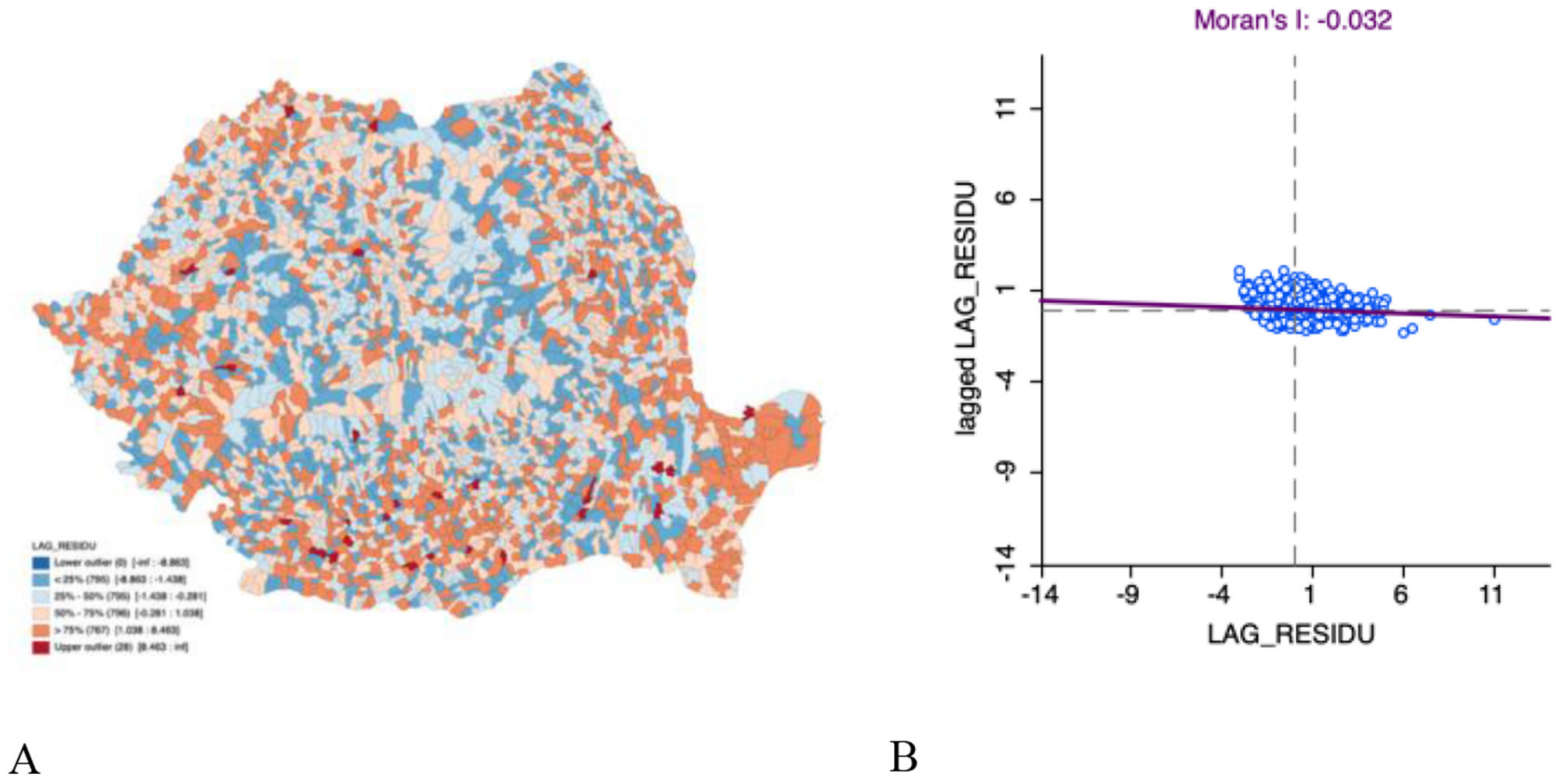
**(A)** Residual choropleth for total incidence of TB (2015–2021) for the spatial lag regression model; **(B)** Global I Moran statistic for total incidence of TB (2015–2021) residuals scatter plot.

Figure 3A presents a choropleth map for the total incidence of TB (2015–2021) residuals for the spatial lag regression model. The residuals appear to be randomly scattered across Romania, suggesting no spatial autocorrelation. Figure 3B displays a scatter plot from the Global I-Moran analysis of the total incidence of TB between 2015 and 2021 residuals. The Moran I is−0.032, further suggesting that the residuals are randomly distributed in space with no meaningful spatial clustering. Based on this analysis, the spatial lag model is the most appropriate as spatial dependence is accounted for. All analyses involving the spatial lag regression model were performed in GeoDa.

## 3 Results

### 3.1 Geospatial descriptive statistics of TB in Romania

**Figure 4:**
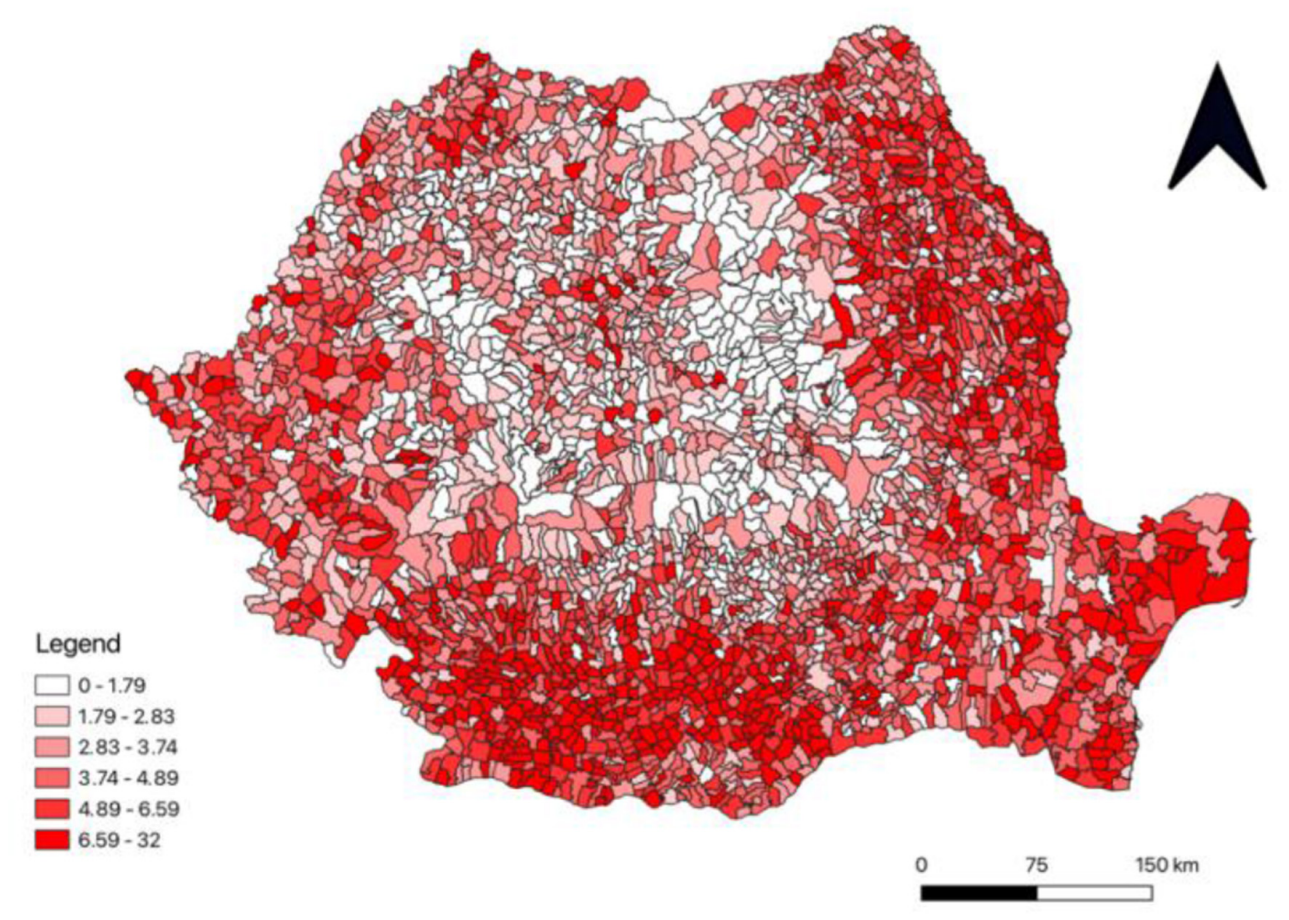
The total incidence of tuberculosis cases between 2015-2021 in Romania.

The spatial distribution of the total incidence of TB cases in Romania, between 2015 and 2021 highlights disparities throughout the country (Figure 4), the highest concentrations are observed in the regions of Oltenia, Muntenia, Dobrogea and Basarabia. There are also some sporadic groups in the Banat region. Localized clusters are present in these regions with higher levels of incidence, ranging from 6.59 to 32 per 1,000 inhabitants. The central and northern areas of Romania have lower tuberculosis incidence values, especially in the region of Moldova.

**Figure 5:**
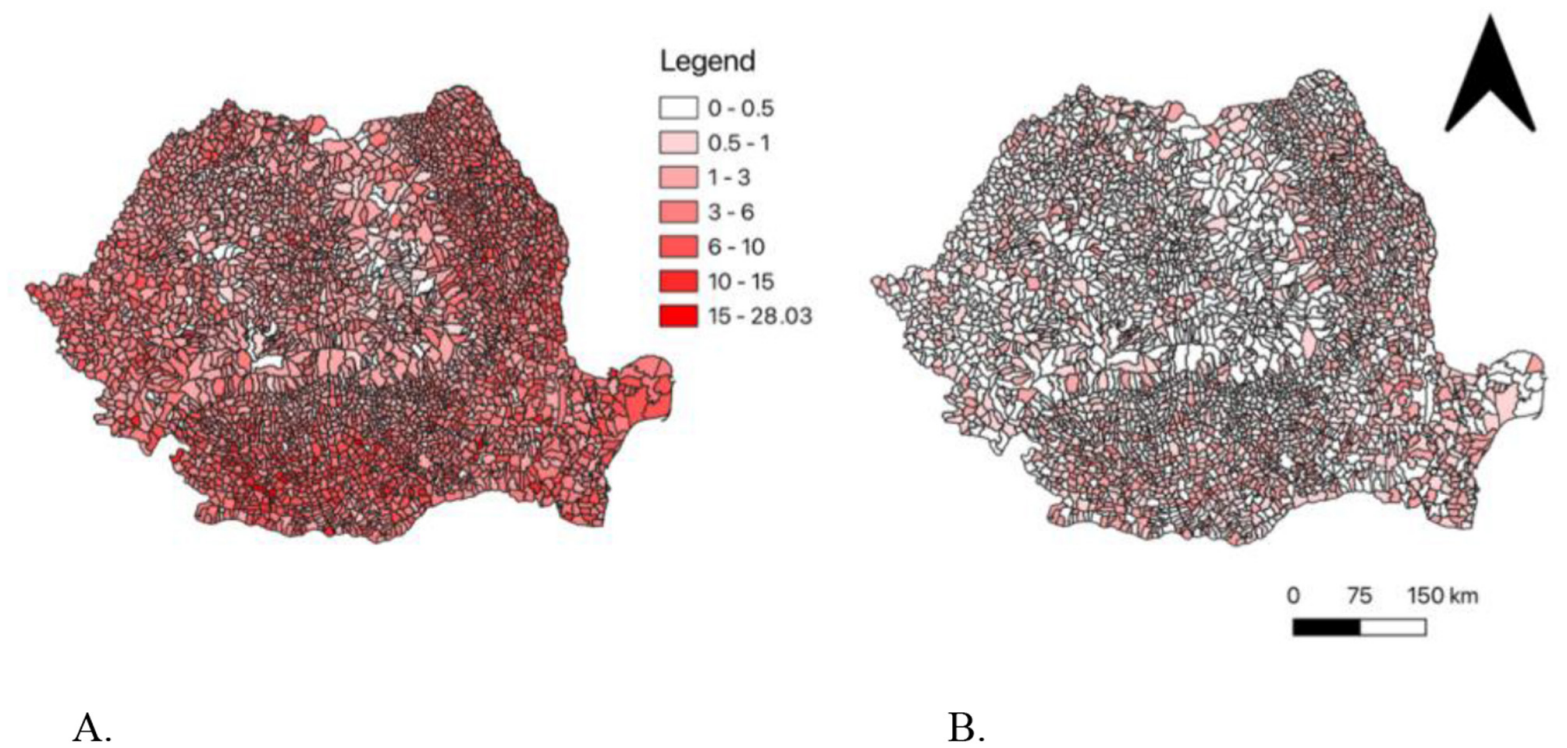
**(A)** The total incidence of pulmonary tuberculosis cases in Romania from 2015–2021; **(B)** The total incidence of non-pulmonary tuberculosis cases in Romania from 2015–2021.

Disaggregated by the affected organ, the total number of pulmonary TB and non-pulmonary TB is compared using the same scale. Pulmonary TB is more incident and spatially clustered than non-pulmonary TB across Romania (5). Pulmonary TB is more concentrated in the Oltenia, Muntenia, Basarabia, and some of the Dobrogea regions of Romania, with rates ranging widely (Figure 5A). Higher rates of pulmonary TB in Romania range from 15 to 28.03 per 1,000 population (Figure 5A). There are clear spatial clusters of pulmonary TB in Romania, particularly in the southern and eastern areas of the country. Overall, non-pulmonary TB presents a much lower incidence compared to pulmonary TB. Many regions of Romania show lower incidence rates of non-pulmonary TB across the country (0–0.5 and 0.5–1 per 1,000 population; Figure 5B). Higher rates of non-pulmonary TB in Romania are less important than in pulmonary TB, and spatial clustering is much less important compared to non-pulmonary TB.

**Figure 6:**
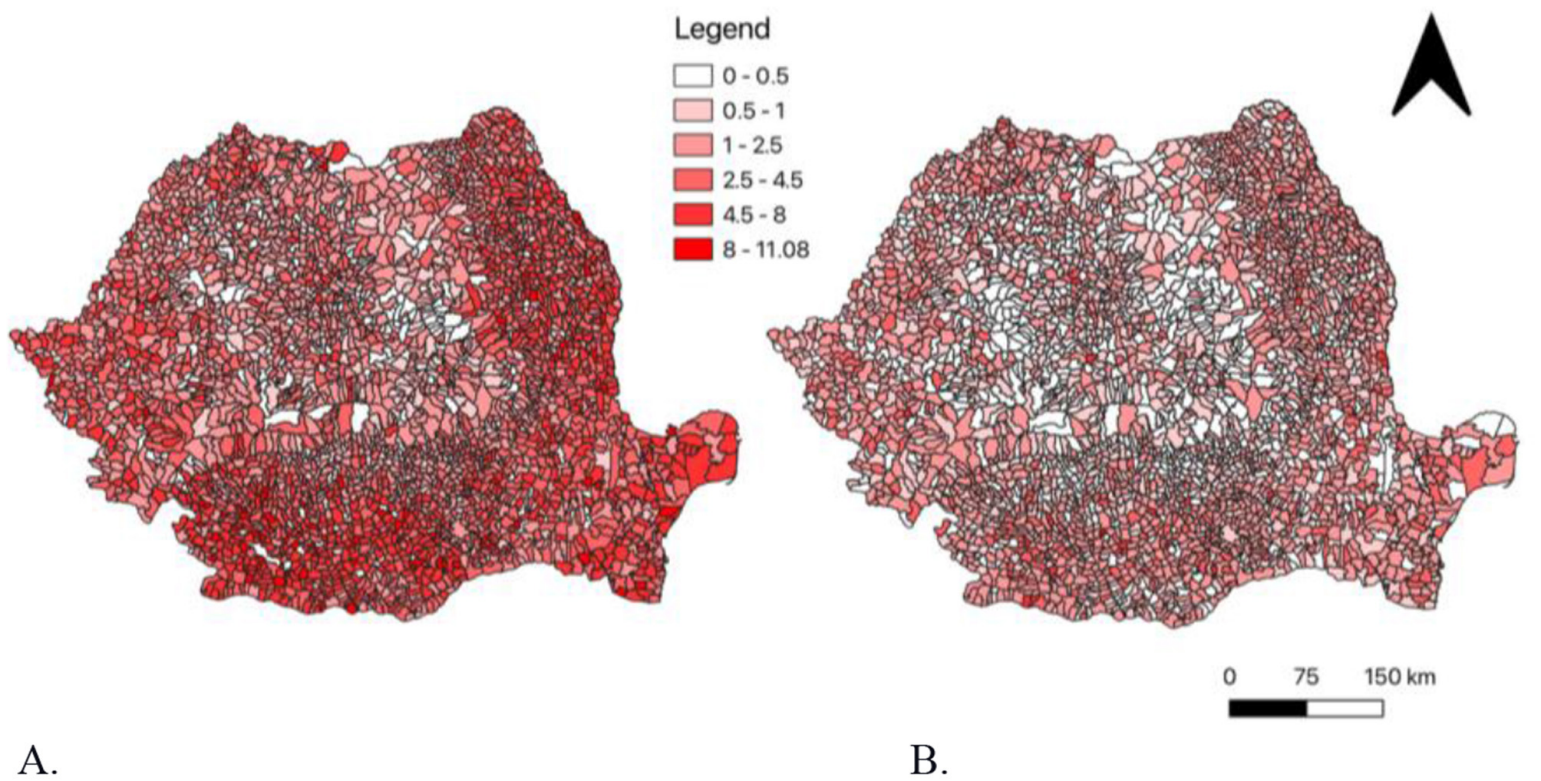
**(A)** The total incidence of tuberculosis cases among males in Romania from 2015–2021; **(B)** The total incidence of tuberculosis cases among Females in Romania from 2015–2021.

The total number of TB is also disaggregated by sex. The total cases of TB among males is compared to that of females in Figure using the same scale. The spatial distribution of TB cases among males is greater than that of females (Figure 6). Elevated frecvence rates of TB among males are most prominent in the Oltenia, eastern Muntenia, Dobrogea, Basarabia, and sporadically in the Banat regions of Romania. Romania (Figure 6A). Notably, areas in the Oltenia, eastern Muntenia, and Brasarabia regions show a frecvence rates of 8–11.08 per 1,000 population among males (Figure 6A). There are visible clusters of regions that have higher TB incidence among males. Females have an overall lower frequency rate of TB in Romania compared to males. There are a few high- clusters present among females, resulting in spatial clustering that is not as prominent (Figure 6B). Most regions in Romania have TB frequency rates below 4.5 cases per 1,000 inhabitants (Figure 6B). Moderate levels of TB are present among females in the Oltenia, Muntenia, and Dobrogea regions. Also, the Basarabia, Banat, Crisana, and Maramureş regions are more sporadic regarding clustering of TB frequency rate among females (Figure 6B). There are considerable differences in comparing total TB frequency cases between males and females in Romania with males visibly showing a greater incidence compared to females.

### 3.2 Geospatial correlation matrix

The Pearson correlation matrix of the total incidence of TB between 2015 and 2021 (i_20152021) and PM2.5 levels (aqli_pm25), as well as the percentage of the population that is illiterate (illit_pop), and PCA1 is highlighted in Table 3. The correlation matrix displays the strength and significance of potential linear relationships between the total incidence of TB from 2015 to 2021 and three explanatory variables: PM2.5 levels, the percentage of the population that is illiterate, and PCA1, a hybrid indicator of living conditions. There is a moderate positive correlation between total incidence of TB and PM2.5 levels (*r* = 0.261, *p* < 0.0001). Higher PM2.5 pollution levels are correlated with higher TB incidence areas in Romania. There is a weak positive correlation between total TB incidence and the percentage of the population that is illiterate (*r* = 0.102, *p* < 0.0001). As the correlation coefficient is weak between total TB incidence and the percentage of the population that is illiterate, further research is needed to understand this correlation fully. There is a moderate positive correlation between total TB incidence and PCA1 (*r* = 0.338, *p* < 0.0001), indicating that the composite living condition indicator from the PCA has a notable association with TB incidence. All reported correlations are statistically significant, as indicated by *p*-values of *p* < 0.0001 for all highlighted associations with the total incidence of TB between 2015 and 2021.

**Table 3:** Pearson correlation matrix of total incidence of TB between 2015–2021 (i_20152021) and three explanatory variables: (aqli_pm25, illit_pop, and PCA1).

**Matrix**	**i_2015-2021 total incidence of TB**	**aqli_pm25 average PM2.5 levels between 2015-2021**	**illit_pop the percentage of illiterate population**	**PCA1**
i_20152021 total incidence of TB	1.00000	0.26084	0.10207	0.33832
		<0.0001	<0.0001	<0.0001
aqli_pm25 average PM2.5 levels between 2015-2021	0.26084	1.00000	0.04364	0.11449
	<0.0001		<0.0001	<0.0001
illit_pop the percentage of illiterate population	0.10207	0.04364	1.00000	0.26746
	<0.0001	<0.0001		<0.0001
PCA1	0.33832	0.11449	0.26746	1.00000
	<0.0001	<0.0001	<0.0001	

Pearson correlation coefficients, N = 3,181; Prob > |r| under H0 Rho = 0.

**Figure 7:**
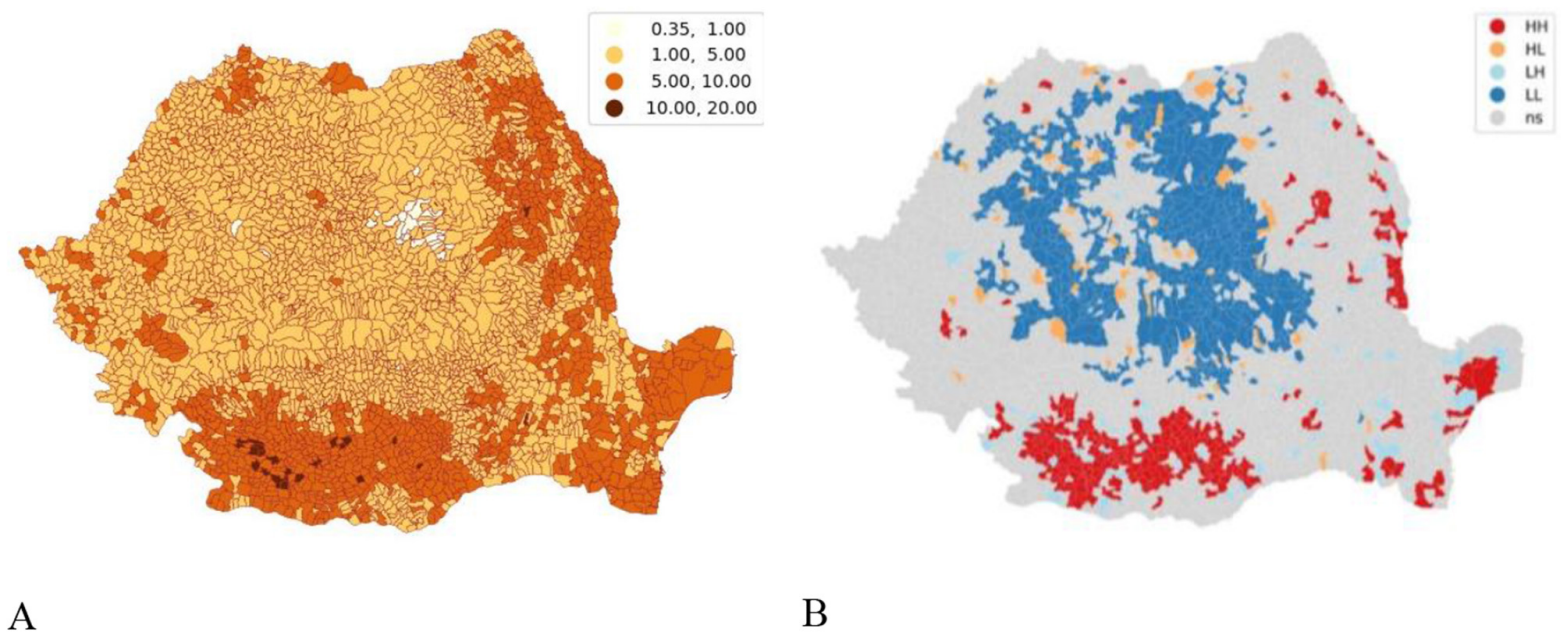
Spatial clustering. **(A)** Spatial autocorrelation outliers of cumulative TB frequency (2015–2021); **(B)** Local Moran spatial clustering. *Source*: *GeoHealth*, spatio-temporal pattern of tuberculosis distribution in Romania and particulate matter pollution association with risk of infection, 2024 (5).

Spatial clustering of total TB case incidence highlights types of clusters at national level, thus high-high clusters are validated in the Oltenia and eastern Muntenia regions and sporadically in the Dobrogea and Basarabia regions. Low-low regions are clustered in the Bucovina, Moldova, and parts of the Transilvania regions of Romania (Figure 7B). High-high and low-low regions make TB sensitive in that particular region. A manual defined legend was used to locate TB areas that are outliers to highlight areas with < 1 case per 1,000 population and areas with more than 10 cases per 1,000 population between 2015 and 2021 (Figure 7A). Through qualitative analysis as such, the causes or conditions for the occurrence/non-occurrence of TB will be able to pinpoint outlier communities (Figure 7A) (5).

**Figure 8:**
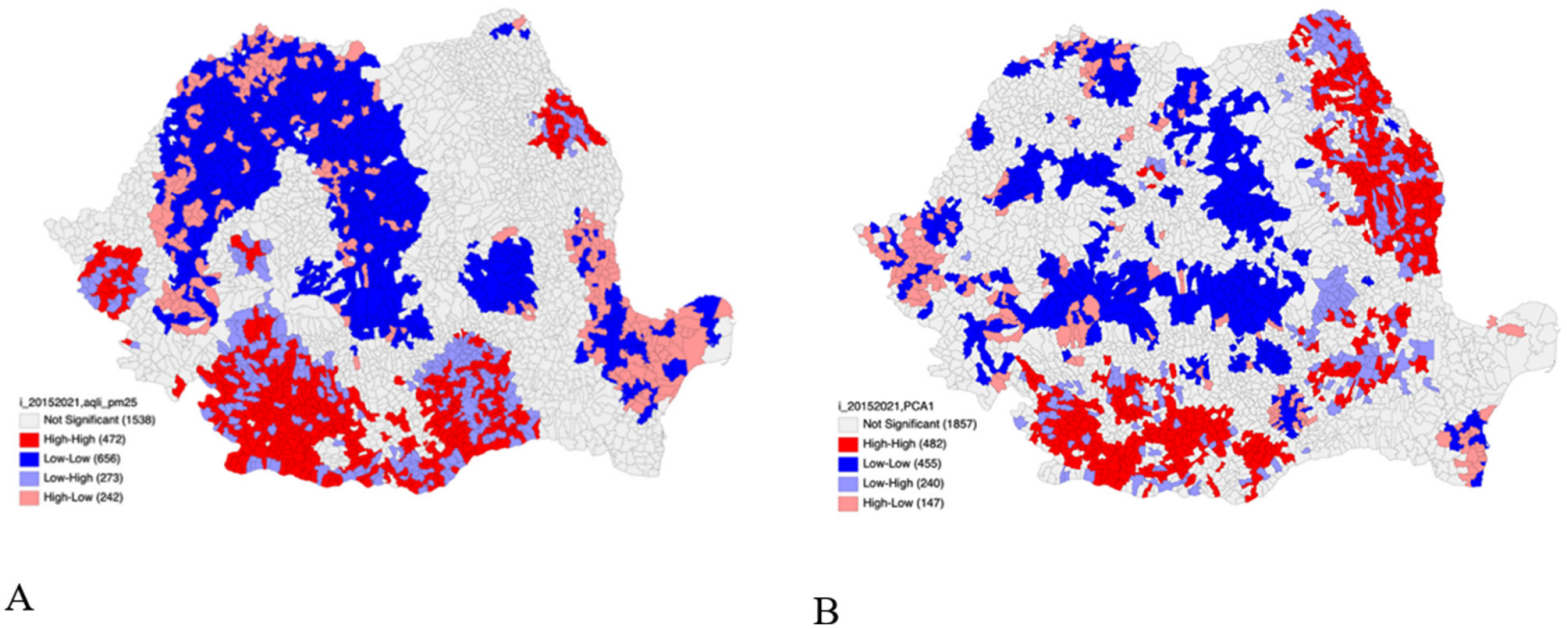
**(A)** Bivariate Moran's I: total incidence of TB (2015–2021) and PM2.5 levels cluster map; **(B)** Bivariate Moran's I: total incidence of TB (2015–2021) and PCA1 cluster map.

A geospatial bivariate analysis was performed between the total incidence of TB and two exploratory variables, PM2.5 levels and PCA1, to investigate the relationship between these variables (Figure 8). The bivariate analysis between the total incidence of TB from 2015 to 2021 and PM2.5 levels reveals numerous areas throughout the country where local clusters are present. The cluster map in Figure 8A displays the spatial bivariate clustering of the total incidence of TB and PM2.5 levels. There are 472 UATs with high-high clusters, shown in red. High-high areas are hotspot areas throughout Romania, characterized by high levels of TB incidence and surrounding neighbors with high levels of PM2.5 pollution. In the bivariate analysis, high-high regions are locally clustered in the Oltenia and Muntenia regions of the country. Two new clusters appear in the Timişoara and Iaşi regions. These new clusters are sensitive to the bivariate relationship between TB incidence and PM2.5 levels.

These regions are new areas that are sensitive to the correlation between total TB incidence and PM2.5 levels during the time period of 2015–2021. There are 656 low-low clusters between total TB incidence and PM2.5 levels displayed in blue. These are areas with both low TB incidence and lower PM2.5 levels. Low-low spots are concentrated in the southern Moldova, Maramureş, and parts of the Cri?ana and Transilvania regions. The cluster map in Figure 8A reveals 273 low-high and 242 high-low spots in Romania. These are outlier areas where TB incidence and PM2.5 levels differ. For example, low-high areas are present when there is an area with low levels of TB incidence surrounded by high levels of PM2.5 pollution. There are also areas in Romania where there is a lack of spatial correlation between TB incidence and PM2.5 levels.

### 3.3 Geospatial bivariate exploratory analysis

The bivariate analysis between the total incidence of TB from 2015 to 2021 and PCA1 reveals numerous areas throughout the country where local clusters are present. The cluster map in Figure 8B displays the spatial bivariate clustering of the total incidence of TB and PCA1. There are 482 UATs that have high-high clusters shown in red. High-high regions are locally clustered in the Oltenia, Moldova, and eastern Muntenia regions of Romania. These are areas that exhibit high TB incidence and high values of the hybrid living condition indicator. There are 455 low-low clusters between total TB incidence and PCA1, shown in blue. These are areas that show low TB incidence is locally clustered with low areas of PCA1. Low-low regions are sporadic in the Moldova, Transilvania, and Maramureş regions of Romania. In the bivariate analysis, between TB incidence and PCA1 (Figure 8B), a more prominent clustering is present the Basarabia region compared to the univariate analysis of total incidence of TB in Figure 7B. This region is sensitive to the correlation between TB incidence and PCA1. The cluster map in Figure 8B reveals 240 low-high and 147 high-low spots in Romania. These are outlier areas where the TB incidence and PCA1 values differ. For example, low-high areas are present when there is an area with low levels of TB incidence surrounded by better living conditions (higher levels of PCA1). High-low and low-high areas at the UAT level are not uniform across Romania and may be influenced by other living conditions not captured in PCA1. There are also areas in Romania where there is a lack of spatial correlation between TB incidence and PCA1.

### 3.4 Geospatial lag regression analysis

A spatial lag regression model was estimated to assess the association between total TB incidence between 2015 and 2021 and key explanatory variables: PM2.5 levels (aqli_pm25), the percentage of the population that is illiterate (illit_pop), and a hybrid living condition variable (PCA1). This model accounts for spatial dependence. The spatial lag coefficient (Rho = 0.436, *p* < 0.0001) is positive and significant. This confirms spatial autocorrelation that neighboring regions influence TB incidence in one UAT. PM2.5 levels showed a statistically significant positive association with TB incidence (β = 0.225, *p* < 0.0001). Higher PM2.5 levels may be associated with increased TB incidence in Romania. PCA1 is also positively and statistically significantly associated with TB incidence (β = 0.314, *p* < 0.0001). Living conditions in Romania may be associated with a higher TB incidence burden. The percentage of the population that is illiterate was not a statistically significant predictor in the model (β = −0.067, *p* = 0.976). Illiteracy does not have a geospatial significant impact on TB incidence in Romania once other socio-economic and environmental indicators are accounted for. The spatial lag model explains 28.4% of the variance in TB incidence between 2015 and 2021 across UATs. The likelihood ratio test for spatial dependence was statistically significant (LR = 373.62, *p* < 0.0001), emphasizing that the spatial lag model was an appropriate choice in this analysis. The likelihood ratio test also proves that spatial autocorrelation of TB incidence is present in Romania. The results from the Breusch-Pagan test for heteroskedasticity were statistically significant (*p* < 0.0001), suggesting non-constant variance in the residuals.

## 4 Discussion and conclusions

This research aims to highlight the determinants associated with the increasing incidence of tuberculosis. Modern epidemiology emphasizes that the development of an infectious disease is made possible by a combination of factors related not only to the etiological agent involved but also to the social and genetic determinism of the population. Tuberculosis in Romania, although the number of cases has progressively decreased in the last 20 years, through structural programs implemented by the Romanian state, there are some particularities of the incidence, especially in certain geographical regions of the country (Figure 4). However, in conditions of unitary measures at the level of the national surveillance and control program, the significant differences in the frequency of tuberculosis cases require an extended analysis of the socio-economic conditions, which are developed differently in the regions that make up Romania. On the other hand, the territorial-administrative organization has other particularities, which required the analysis model to be shared accordingly.

A longitudinal study conducted between 2005–2015, which included 48 low- and middle-income countries and 68 high- and upper-middle-income countries and aimed to estimate associations between national TB incidence rates and 13 social determinants of health, concluded that low- and upper-middle-income countries have the highest TB incidence rates, correlating with increased prevalence of diabetes and alcohol consumption (56). Compared to this study, our research aims at evaluating the territorial administrative units, and may open new perspectives on the health policies Romania needs.

Starting from the differences related to the different pathophysiological mechanisms of pulmonary and extrapulmonary tuberculosis, the analysis carried out in our research and highlighted in Figure 5 reveals that extrapulmonary tuberculosis has a relatively uniform distribution, bringing a small number of cases compared to pulmonary tuberculosis which creates specific clusters in the eastern and southern parts of Romania (16). This leads us to conclude that the factor that induces immunosuppression, and that favors the mechanism of endogenous activation of status-specific dormant twins of primary tuberculosis infection, is relatively common to the entire population (56).

In Wetzstein's study, a number of hypotheses are raised addressing a possible increased risk of extrapulmonary tuberculosis in relation to geographical factors, with the study indicating increased incidence due to late diagnosis, impaired host immune system, but also possible virulence factors of MTB correlated with relevant comorbidities (57).

The high incidence of pulmonary tuberculosis in certain geographical regions in Romania is probably related to the difficult access to health care, due to the uneven distribution of doctors or limited health services in certain regions of the country, a situation also evidenced in rural Henan, China, where the number of pulmonary TB cases is higher in areas farther away from health centers (58).

The observed higher pulmonary TB incidence among males warrant further exploration. Potential explanations include occupational exposures more common among men, for example, industrial or agricultural workers more susceptible to exposure. Other considerations could be linked to behavioral factors such as smoking patterns or delays in seeking medical attention. This study lacked individual-level clinical and behavioral data exploration, limiting deeper investigation. Future research combining epidemiological, clinical, socio-economic, and even behavioral data is helpful to identify patterns and make tailored public health interventions.

With regard to the link between the increased frequency of tuberculosis in males identified in our study, shown graphically in Figure 6, it seems to be correlated with occupational exposure, which implies heavier work performed by men, but also by specific behavioral factors. Horton estimates that TB prevalence is significantly higher among men than among women in low- and middle-income countries, and there is strong evidence that men are disadvantaged by excessive seeking and/or delayed access to TB care (59).

However, recent studies point to possible hormonal and genetic mechanisms that may modulate innate and adaptive immune responses in males and females, leading to sex differences in susceptibility to disease (60).

Our study shows a heterogeneous interaction between tuberculosis incidence, living conditions, air pollution and education indicators in Romania. What we believe should require the authorities to re-evaluate the framework to create geographically targeted public health interventions according to the administrative territorial units identified as high risk. Poor air quality may be linked to an increased risk of tuberculosis in the high-high clusters. The low-high and high-high-low outliers suggest that there are other local factors likely influencing the incidence of tuberculosis in addition to air pollution. Pollution has an impact on tuberculosis in the main areas of urban development and in areas with limited access to services/socio-economic facilities/worsened living conditions. There is a new area sensitive to the correlation between tuberculosis incidence and PM2.5 levels (Timisoara and Iasi regions), as shown in Figure 8A. Moreover, another study carried out in Romania indicates a correlation between pollution levels and increased incidence of respiratory diseases (61).

In our study we were able to highlight areas with high risk of tuberculosis, Figure 8B, correlated with poor living conditions and poor access to services/facilities located in the southern and eastern regions of Romania. Low-low areas suggest that better living conditions coincide with a lower burden of TB in the northern and central regions of Romania. High-low and low-high regions suggest that socio-economic characteristics at the UAT levels are not uniform across the country and may be influenced by other indicators that the PCA1 hybrid indicator of living conditions does not capture. The Basarabia region is more sensitive to the bivariate analysis between TB incidence and PCA1, as this area shows a large-large clustering. Compared to the univariate analysis of TB incidence, this region does not have nearly as much large-major clustering, as shown in Figure 8B. National studies indicate a different incidence of TB in vulnerable population groups in the same geographic region, which makes the current study to bring new insights that need to be addressed in public health policies that create effective solutions to reduce poverty and social exclusion, estimated in 2024 according to Eurostat at an average of 27.9% (62, 63).

The geographic clustering of pulmonary tuberculosis (PTB) suggests a distinct epidemiologic pattern, also found in other published studies. In East Gojjam Zone, Ethiopia, PTB clustering has been associated with rural residence, distance from health facilities, and poor training of tuberculosis services (64). In Hubei province, China, PTB cases were spatially clustered, with high-risk areas identified in the southwest and southeast regions (65). Seasonal patterns and periodicity in PTB incidence were observed, with peaks in late spring and early summer (65). Special attention should also be paid to specific geographical areas such as urban catchment areas or mining basins where companies considered as major polluters have operated (66–70).

The study provides new insights into the association of increased incidences of PM 2.5 pollutant levels and living conditions with the increased risk of people in these regions to get tuberculosis. Table 4 illustrates this link. Another aspect worth mentioning is the lack of statistically significant association between the percentage of illiteracy and the incidence of TB revealed by the current research, although a targeted analysis of vulnerable groups, and of the interventions carried out in the study by Munteanu et al. (71) shows a low level of education among people in vulnerable groups and TB patients.

**Table 4:** Spatial lag regression analysis results.

**Regression**				
**Summary of output**	**Spatial lag model–maximum likelihood estimation**			
Data set	tbc_2015_2021_full2			
Spatial weight	tbc_2015_2021_full2			
Dependent variable	i_20152021	Number of observations	3,181	
Mean dependent var	4.25484	Number of variables	5	
S.D. dependent var	2.78239	Degrees of freedom	3,176	
Lag coeff. (Rho)	0.435627			
*R*-squared	0.284036	Log likelihood	−7,296.09	
Sq. correlation	–	Akaike info criterion	14,602.2	
Sigma-square	5.54278	Schwarz criterion	14,632.5	
S.E of regression	2.35431			
**Variable**	**Coefficient**	**std.error**	***z*** **-value**	**Probability**
W_i_20152021	0.435627	0.0224235	19.4273	0.00000
Constant	−1.01153	0.431196	−2.34588	0.01898
aqli_pm25	0.225037	0.0295927	7.60447	0.00000
PCA1	0.313559	0.0240231	13.0524	0.00000
illit_pop	−0.0669543	2.25135	−0.0297396	0.97627
**Regression diagnostics**				
**Diagnostics for heteroskedasticity**				
**Random coefficients**				
**Test**	**DF**	**Value**	**Prob**	
Breusch-Pagan test	3	86.0687	0.00000	
Diagnostics for spatial dependence				
Spatial lag dependence for weight matrix	tbc_2015_2021_full2			
Test	DF	Value	Prob	
Likelihood ratio test	1	373.6227	0.00000	
**Coefficients variance matrix**				
**Constant**	**aqli_pm25**	**PCA1**	**illit_pop**	**W_i_20152021**
0.185930	−0.012346	0.000976	−0.092758	0.001141
−0.012346	0.000876	−0.000003	−0.000695	−0.000215
0.000976	−0.000003	0.000577	−0.013586	−0.000154
−0.092758	−0.000695	−0.013586	5.068579	−0.000379
0.001141	−0.000215	−0.000154	−0.000379	0.000503

Although our analysis revealed a weak positive correlation between illiteracy rates and TB incidence in bivariate analysis, illiteracy was not a significant predictor in the spatial lag regression model. This could be explained by a few factors. First, illiteracy could be intercorrelated with other socio-economic variables that the PCA1 hybrid living condition indicator captured. Second, the impact of educational attainment on the risk of TB can be context-specific. Previous literature reports mixed findings (72). In some settings, lower educational attainment strongly predicts TB while in other studies, the effect lowers when other socio-economic and environmental factors are considered. Differences in data availability, regional location, and potential unmeasured confounders should also be considered. Future studies should integrate more in-depth socio-economic data to determine the role educational attainment has on TB incidence.

The aggregation level (UAT) in Romania may account for local heterogeneity in the impact illiteracy has on TB risk. Alternative analytic techniques could help to better clarify this relationship.

We believe that this hypothesis needs further study, because several studies indicate this TB–education correlation. However, heteroscedasticity is a limitation in the spatial lag model, this is a common limitation in spatial modeling, but it does not invalidate the model. GeoDa does not have this. power, further analysis is needed in the future The spatial lag model effectively accounts for spatial autocorrelation which is significant in general and explains significant variation. The residuals show no spatial clustering (Moran's I is close to zero), so the spatial structure is well-captured. Certainly, there are limitations of our study which are given by the potential for heteroskedasticity in the spatial delay model, the use of age standardization being well-recognized in the scientific world, we choose not to use it, because it brings errors in the data analysis, being dependent on the standard population, and not reflecting the real risk of tuberculosis. Additionally, the spatial lag regression model explained ~28.4% of the variation in TB incidence rates across Romania. While the model captures significant predictors, a large portion of variance remains unaccounted for. Other socio-economic and environmental indicators that were no available for analysis in this study should include such factors.

The current research requires further processing steps to include age-standardized incidence rates, as age is usually a good determinant of TB risk and can vary substantially between different age groups.

## Data Availability

The raw data supporting the conclusions of this article will be made available by the authors, without undue reservation.
